# 4-[2-(4-Bromo­phen­yl)hydrazinyl­idene]-3-methyl-5-oxo-4,5-dihydro-1*H*-pyrazole-1-carbothio­amide

**DOI:** 10.1107/S1600536811034726

**Published:** 2011-09-14

**Authors:** Hoong-Kun Fun, Madhukar Hemamalini, Shobhitha Shetty, BalaKrishna Kalluraya

**Affiliations:** a X-ray Crystallography Unit, School of Physics, Universiti Sains Malaysia, 11800 USM, Penang, Malaysia; b Department of Studies in Chemistry, Mangalore University, Mangalagangotri, Mangalore 574 199, India

## Abstract

In the title compound, C_11_H_10_BrN_5_OS, the approximately planar pyrazole ring [maximum deviation = 0.014 (2) Å] forms a dihedral angle of 5.49 (13)° with the benzene ring. An intra­molecular N—H⋯O hydrogen bond generates an *S*(6) ring motif. In the crystal, mol­ecules are linked through inter­molecular N—H⋯S and N—H⋯O hydrogen bonds, forming a two-dimensional network parallel to (100). A short Br⋯Br contact of 3.5114 (6) Å is also observed.

## Related literature

For details and applications of pyrazole compounds, see: Isloor *et al.* (2009 ▶); Rai *et al.* (2008 ▶) Bradbury & Pucci (2008 ▶); Girisha *et al.* (2010 ▶). For standard bond-length data, see: Allen *et al.* (1987 ▶). For hydrogen-bond motifs, see: Bernstein *et al.* (1995 ▶).
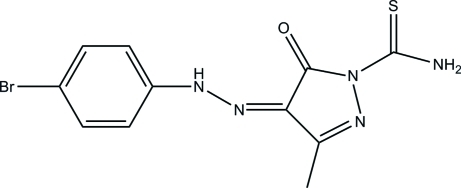


## Experimental

### 

#### Crystal data

C_11_H_10_BrN_5_OS
*M* *_r_* = 340.21
Monoclinic, 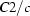

*a* = 25.6080 (18) Å
*b* = 11.6686 (8) Å
*c* = 9.0823 (6) Å
β = 98.907 (2)°
*V* = 2681.2 (3) Å^3^
*Z* = 8
Mo *K*α radiation
μ = 3.22 mm^−1^
*T* = 296 K
0.48 × 0.33 × 0.17 mm

#### Data collection

Bruker APEXII DUO CCD area-detector diffractometer
Absorption correction: multi-scan (*SADABS*; Bruker, 2009 ▶) *T*_min_ = 0.306, *T*_max_ = 0.609
15576 measured reflections
3869 independent reflections
2776 reflections with *I* > 2σ(*I*)
*R*_int_ = 0.034

#### Refinement

*R*[*F* ^2^ > 2σ(*F* ^2^)] = 0.040
*wR*(*F* ^2^) = 0.127
*S* = 1.03
3869 reflections
185 parameters
H atoms treated by a mixture of independent and constrained refinement
Δρ_max_ = 0.46 e Å^−3^
Δρ_min_ = −0.75 e Å^−3^

### 

Data collection: *APEX2* (Bruker, 2009 ▶); cell refinement: *SAINT* (Bruker, 2009 ▶); data reduction: *SAINT*; program(s) used to solve structure: *SHELXTL* (Sheldrick, 2008 ▶); program(s) used to refine structure: *SHELXTL*; molecular graphics: *SHELXTL* and *PLATON* (Spek, 2009 ▶); software used to prepare material for publication: *SHELXTL* and *PLATON*.

## Supplementary Material

Crystal structure: contains datablock(s) global, I. DOI: 10.1107/S1600536811034726/lh5323sup1.cif

Structure factors: contains datablock(s) I. DOI: 10.1107/S1600536811034726/lh5323Isup2.hkl

Supplementary material file. DOI: 10.1107/S1600536811034726/lh5323Isup3.cml

Additional supplementary materials:  crystallographic information; 3D view; checkCIF report

## Figures and Tables

**Table 1:** Hydrogen-bond geometry (Å, °)

*D*—H⋯*A*	*D*—H	H⋯*A*	*D*⋯*A*	*D*—H⋯*A*
N4—H1*N*4⋯O1	0.82 (4)	2.27 (4)	2.788 (3)	121 (3)
N5—H1*N*5⋯S1^i^	0.80 (4)	2.84 (4)	3.522 (2)	144 (3)
N5—H2*N*5⋯O1^ii^	0.82 (3)	2.11 (4)	2.925 (3)	175 (4)

Symmetry codes: (i) 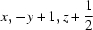; (ii) 
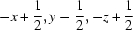
.

**Fig. 1.**
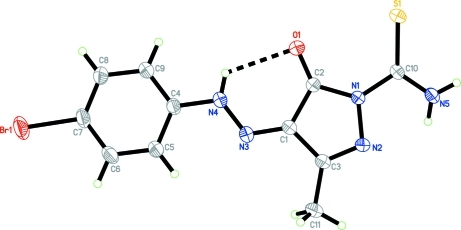
The molecular structure of title compound, showing 50% probability displacement ellipsoids. An intramolecular hydrogen bond is shown by a dashed line.

**Fig. 2.**
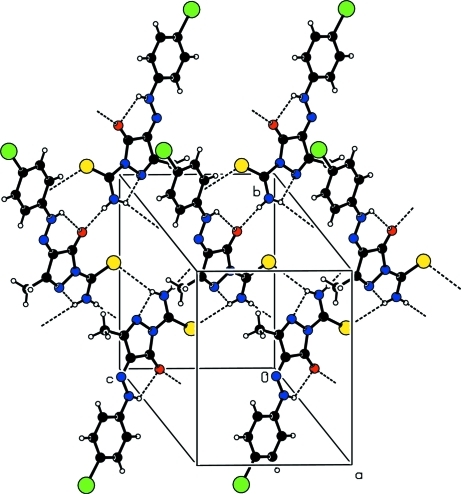
The crystal packing of (I) with hydrogen bonds shown as dashed lines.

## supplementary crystallographic information

### Comment

The pyrazole ring is a prominent structural moiety found in numerous pharmaceutically active compounds. This is mainly due to the easy preparation and the important pharmacological activity. Therefore, the synthesis and selective functionalization of pyrazoles have been the focus of active research area over the years (Isloor *et al.*, 2009). Pyrazoles have been reported to possess antibacterial activity (Rai *et al.*, 2008), and inhibitor activity against DNA gyrase and topoisomerase IV at their respective ATP-binding sites (Bradbury & Pucci, 2008). Moreover, pyrazole-containing compounds have received considerable attention owing to their diverse chemotherapeutic potentials including versatile anti-inflammatory and antimicrobial activities (Girisha *et al.*, 2010).  The synthetic route followed for obtaining the title compound involves the diazotization of substituted anilines to give the diazonium salts followed by coupling with ethyl acetoacetate in the presence of sodium acetate to give the corresponding oxobutanoate which on further reaction  with thiosemicarbazide in acetic acid gave the required thioamides.

The asymmetric unit of the title compound (I) is shown in Fig. 1. The pyrazole (N1,N2/C1–C3) ring is approximately planar, with a maximum deviation of 0.014 (2) Å for atom N1.  The dihedral angle between the benzene (C4–C9) ring and the pyrazole (N1,N2/C1–C3) ring is 5.49 (13)°. An intramolecular N4—H1N4···O1 hydrogen bond generates an *S*(6) ring motif (Bernstein *et al.*, 1995). The bond lengths (Allen *et al.*, 1987) and angles are within normal ranges.

In the crystal structure (Fig. 2) molecules are linked through intermolecular N5—H1N5···S1^i^ and N5—H2N5···O1^ii^ hydrogen bonds (Table 1) forming a two-dimensional network parallel to (1 0 0). A short Br···Br contact of 3.5114 (6) Å is also observed.

### Experimental

To a solution of ethyl-2-[(4-bromophenyl)hydrazono]-3-oxobutanoate (0.01 mol) dissolved in glacial acetic acid (20 ml), a solution of thiosemicarbazide (0.02 mol) in glacial acetic acid (25 ml) was added and the mixture was refluxed for 4 h. This was cooled and allowed to stand overnight. The solid product which separated out was filtered and dried. It was then recrystallized from ethanol. Crystals suitable for X-ray analysis were obtained by slow evaporation of a solution of (I) in a 1:2 mixture of DMF and ethanol.

### Refinement

Atoms H1N4, H1N5 and H2N5 were located in difference Fourier maps and refined freely [N–H = 0.81 (4)–0.82 (3) Å]. The remaining H atoms were positioned geometrically [C–H = 0.93 or 0.96 Å] and were refined using a riding model, with *U*_iso_(H) = 1.2 or 1.5 *U*_eq_(C).  A rotating group model was applied to the methyl groups.

### Crystal data

**Table:**

C_11_H_10_BrN_5_OS	*F*(000) = 1360
*M**_r_* = 340.21	*D*_x_ = 1.686 Mg m^−^^3^
Monoclinic, *C*2/*c*	Mo *K*α radiation, λ = 0.71073 Å
Hall symbol: -C 2yc	Cell parameters from 4204 reflections
*a* = 25.6080 (18) Å	θ = 2.9–27.8°
*b* = 11.6686 (8) Å	µ = 3.22 mm^−^^1^
*c* = 9.0823 (6) Å	*T* = 296 K
β = 98.907 (2)°	Slab, orange
*V* = 2681.2 (3) Å^3^	0.48 × 0.33 × 0.17 mm
*Z* = 8	

### Data collection

**Table:**

Bruker APEXII DUO CCD area-detector diffractometer	3869 independent reflections
Radiation source: fine-focus sealed tube	2776 reflections with *I* > 2σ(*I*)
graphite	*R*_int_ = 0.034
φ and ω scans	θ_max_ = 30.0°, θ_min_ = 2.9°
Absorption correction: multi-scan (*SADABS*; Bruker, 2009)	*h* = −36→36
*T*_min_ = 0.306, *T*_max_ = 0.609	*k* = −16→14
15576 measured reflections	*l* = −12→12

### Refinement

**Table:**

Refinement on *F*^2^	Primary atom site location: structure-invariant direct methods
Least-squares matrix: full	Secondary atom site location: difference Fourier map
*R*[*F*^2^ > 2σ(*F*^2^)] = 0.040	Hydrogen site location: inferred from neighbouring sites
*wR*(*F*^2^) = 0.127	H atoms treated by a mixture of independent and constrained refinement
*S* = 1.03	*w* = 1/[σ^2^(*F*_o_^2^) + (0.0646*P*)^2^ + 2.3027*P*] where *P* = (*F*_o_^2^ + 2*F*_c_^2^)/3
3869 reflections	(Δ/σ)_max_ = 0.001
185 parameters	Δρ_max_ = 0.46 e Å^−^^3^
0 restraints	Δρ_min_ = −0.75 e Å^−^^3^

### Special details

**Table:**

Geometry. All s.u.'s (except the s.u. in the dihedral angle between two l.s. planes) are estimated using the full covariance matrix. The cell s.u.'s are taken into account individually in the estimation of s.u.'s in distances, angles and torsion angles; correlations between s.u.'s in cell parameters are only used when they are defined by crystal symmetry. An approximate (isotropic) treatment of cell s.u.'s is used for estimating s.u.'s involving l.s. planes.
Refinement. Refinement of F^2^ against ALL reflections. The weighted R-factor wR and goodness of fit S are based on F^2^, conventional R-factors R are based on F, with F set to zero for negative F^2^. The threshold expression of F^2^ > 2σ(F^2^) is used only for calculating R-factors(gt) etc. and is not relevant to the choice of reflections for refinement. R-factors based on F^2^ are statistically about twice as large as those based on F, and R- factors based on ALL data will be even larger.

### Fractional atomic coordinates and isotropic or equivalent isotropic displacement parameters (Å^2^)

**Table:**

	*x*	*y*	*z*	*U*_iso_*/*U*_eq_	
S1	0.24780 (3)	0.67093 (6)	0.16175 (6)	0.04971 (18)	
Br1	0.476115 (14)	1.36166 (3)	0.95277 (4)	0.07714 (17)	
O1	0.29888 (8)	0.85438 (14)	0.39141 (19)	0.0476 (4)	
N1	0.30362 (8)	0.65467 (16)	0.43648 (19)	0.0377 (4)	
N2	0.32861 (9)	0.58425 (17)	0.5541 (2)	0.0442 (5)	
N3	0.37576 (9)	0.85410 (17)	0.6771 (2)	0.0433 (4)	
N4	0.36660 (9)	0.95754 (18)	0.6236 (2)	0.0432 (4)	
N5	0.26122 (10)	0.4932 (2)	0.3443 (2)	0.0483 (5)	
C1	0.35129 (9)	0.7684 (2)	0.6042 (2)	0.0399 (5)	
C2	0.31496 (9)	0.77032 (19)	0.4632 (2)	0.0358 (4)	
C3	0.35624 (11)	0.6506 (2)	0.6496 (3)	0.0464 (6)	
C4	0.39352 (9)	1.0510 (2)	0.6975 (2)	0.0398 (5)	
C5	0.43053 (11)	1.0338 (2)	0.8242 (3)	0.0536 (6)	
H5A	0.4388	0.9600	0.8588	0.064*	
C6	0.45488 (11)	1.1273 (3)	0.8982 (3)	0.0581 (7)	
H6A	0.4794	1.1169	0.9840	0.070*	
C7	0.44277 (10)	1.2359 (2)	0.8449 (3)	0.0500 (6)	
C8	0.40611 (11)	1.2539 (2)	0.7174 (3)	0.0517 (6)	
H8A	0.3983	1.3276	0.6818	0.062*	
C9	0.38156 (11)	1.1601 (2)	0.6447 (3)	0.0498 (6)	
H9A	0.3568	1.1705	0.5595	0.060*	
C10	0.27077 (9)	0.6012 (2)	0.3187 (2)	0.0373 (5)	
C11	0.38809 (16)	0.6072 (3)	0.7892 (4)	0.0753 (10)	
H11A	0.3828	0.5261	0.7969	0.113*	
H11B	0.3772	0.6450	0.8733	0.113*	
H11C	0.4248	0.6224	0.7875	0.113*	
H1N4	0.3475 (14)	0.980 (3)	0.548 (4)	0.064 (9)*	
H1N5	0.2727 (15)	0.468 (3)	0.425 (4)	0.077 (11)*	
H2N5	0.2458 (13)	0.456 (3)	0.275 (4)	0.057 (9)*	

### Atomic displacement parameters (Å^2^)

**Table:**

	*U*^11^	*U*^22^	*U*^33^	*U*^12^	*U*^13^	*U*^23^
S1	0.0660 (4)	0.0450 (4)	0.0326 (3)	0.0018 (3)	−0.0098 (2)	0.0007 (2)
Br1	0.0707 (2)	0.0514 (2)	0.0982 (3)	−0.00776 (14)	−0.02193 (18)	−0.02813 (16)
O1	0.0595 (11)	0.0342 (9)	0.0437 (9)	0.0036 (7)	−0.0085 (8)	0.0020 (6)
N1	0.0453 (10)	0.0322 (10)	0.0316 (8)	−0.0025 (7)	−0.0064 (7)	0.0006 (6)
N2	0.0550 (12)	0.0331 (10)	0.0387 (9)	−0.0027 (9)	−0.0111 (8)	0.0047 (7)
N3	0.0467 (11)	0.0381 (11)	0.0420 (10)	−0.0061 (8)	−0.0032 (8)	−0.0022 (7)
N4	0.0470 (11)	0.0362 (11)	0.0422 (10)	−0.0034 (8)	−0.0064 (8)	−0.0040 (8)
N5	0.0642 (14)	0.0411 (12)	0.0341 (9)	−0.0113 (10)	−0.0092 (9)	−0.0014 (8)
C1	0.0442 (12)	0.0366 (12)	0.0350 (9)	−0.0020 (9)	−0.0055 (8)	−0.0002 (8)
C2	0.0408 (11)	0.0326 (11)	0.0327 (9)	0.0001 (9)	0.0018 (8)	−0.0002 (8)
C3	0.0529 (14)	0.0399 (13)	0.0406 (11)	−0.0057 (10)	−0.0112 (10)	0.0037 (9)
C4	0.0388 (11)	0.0379 (12)	0.0411 (10)	−0.0039 (9)	0.0012 (9)	−0.0064 (9)
C5	0.0528 (15)	0.0406 (14)	0.0599 (14)	0.0007 (11)	−0.0151 (11)	−0.0046 (11)
C6	0.0519 (15)	0.0527 (17)	0.0606 (15)	0.0019 (12)	−0.0203 (12)	−0.0123 (12)
C7	0.0441 (13)	0.0413 (14)	0.0609 (14)	−0.0045 (10)	−0.0036 (10)	−0.0158 (11)
C8	0.0554 (15)	0.0351 (13)	0.0600 (14)	−0.0030 (11)	−0.0053 (11)	−0.0044 (10)
C9	0.0531 (15)	0.0413 (14)	0.0492 (12)	−0.0033 (11)	−0.0104 (10)	−0.0018 (10)
C10	0.0412 (11)	0.0388 (12)	0.0301 (9)	−0.0009 (9)	−0.0006 (8)	−0.0038 (8)
C11	0.095 (2)	0.0536 (17)	0.0604 (17)	−0.0116 (17)	−0.0407 (16)	0.0130 (13)

### Geometric parameters (Å, °)

**Table:**

S1—C10	1.667 (2)	C1—C2	1.462 (3)
Br1—C7	1.893 (2)	C3—C11	1.486 (3)
O1—C2	1.214 (3)	C4—C9	1.377 (4)
N1—C2	1.394 (3)	C4—C5	1.387 (3)
N1—C10	1.401 (3)	C5—C6	1.378 (4)
N1—N2	1.420 (3)	C5—H5A	0.9300
N2—C3	1.289 (3)	C6—C7	1.375 (4)
N3—C1	1.304 (3)	C6—H6A	0.9300
N3—N4	1.308 (3)	C7—C8	1.389 (4)
N4—C4	1.404 (3)	C8—C9	1.379 (4)
N4—H1N4	0.82 (3)	C8—H8A	0.9300
N5—C10	1.311 (3)	C9—H9A	0.9300
N5—H1N5	0.81 (4)	C11—H11A	0.9600
N5—H2N5	0.81 (4)	C11—H11B	0.9600
C1—C3	1.435 (3)	C11—H11C	0.9600
			
C2—N1—C10	130.42 (19)	C6—C5—H5A	120.3
C2—N1—N2	111.85 (17)	C4—C5—H5A	120.3
C10—N1—N2	117.67 (18)	C7—C6—C5	119.8 (2)
C3—N2—N1	107.16 (19)	C7—C6—H6A	120.1
C1—N3—N4	118.3 (2)	C5—C6—H6A	120.1
N3—N4—C4	119.5 (2)	C6—C7—C8	121.3 (2)
N3—N4—H1N4	131 (2)	C6—C7—Br1	118.24 (19)
C4—N4—H1N4	110 (2)	C8—C7—Br1	120.5 (2)
C10—N5—H1N5	117 (3)	C9—C8—C7	118.6 (3)
C10—N5—H2N5	117 (2)	C9—C8—H8A	120.7
H1N5—N5—H2N5	125 (4)	C7—C8—H8A	120.7
N3—C1—C3	125.1 (2)	C4—C9—C8	120.4 (2)
N3—C1—C2	128.6 (2)	C4—C9—H9A	119.8
C3—C1—C2	106.37 (19)	C8—C9—H9A	119.8
O1—C2—N1	130.2 (2)	N5—C10—N1	113.5 (2)
O1—C2—C1	126.8 (2)	N5—C10—S1	124.77 (17)
N1—C2—C1	103.00 (18)	N1—C10—S1	121.74 (17)
N2—C3—C1	111.6 (2)	C3—C11—H11A	109.5
N2—C3—C11	122.7 (2)	C3—C11—H11B	109.5
C1—C3—C11	125.7 (2)	H11A—C11—H11B	109.5
C9—C4—C5	120.5 (2)	C3—C11—H11C	109.5
C9—C4—N4	119.0 (2)	H11A—C11—H11C	109.5
C5—C4—N4	120.4 (2)	H11B—C11—H11C	109.5
C6—C5—C4	119.3 (3)		
			
C2—N1—N2—C3	−2.1 (3)	C2—C1—C3—C11	−178.2 (3)
C10—N1—N2—C3	−179.5 (2)	N3—N4—C4—C9	−176.6 (2)
C1—N3—N4—C4	−178.2 (2)	N3—N4—C4—C5	1.5 (4)
N4—N3—C1—C3	−178.7 (2)	C9—C4—C5—C6	0.8 (4)
N4—N3—C1—C2	2.4 (4)	N4—C4—C5—C6	−177.4 (3)
C10—N1—C2—O1	0.6 (4)	C4—C5—C6—C7	−0.8 (5)
N2—N1—C2—O1	−176.3 (2)	C5—C6—C7—C8	0.3 (5)
C10—N1—C2—C1	179.5 (2)	C5—C6—C7—Br1	178.7 (2)
N2—N1—C2—C1	2.6 (2)	C6—C7—C8—C9	0.3 (4)
N3—C1—C2—O1	−4.0 (4)	Br1—C7—C8—C9	−178.1 (2)
C3—C1—C2—O1	176.9 (2)	C5—C4—C9—C8	−0.2 (4)
N3—C1—C2—N1	177.0 (3)	N4—C4—C9—C8	178.0 (2)
C3—C1—C2—N1	−2.0 (3)	C7—C8—C9—C4	−0.4 (4)
N1—N2—C3—C1	0.7 (3)	C2—N1—C10—N5	−167.1 (2)
N1—N2—C3—C11	179.8 (3)	N2—N1—C10—N5	9.7 (3)
N3—C1—C3—N2	−178.2 (3)	C2—N1—C10—S1	13.9 (4)
C2—C1—C3—N2	0.9 (3)	N2—N1—C10—S1	−169.28 (17)
N3—C1—C3—C11	2.6 (5)		

### Hydrogen-bond geometry (Å, °)

**Table:**

*D*—H···*A*	*D*—H	H···*A*	*D*···*A*	*D*—H···*A*
N4—H1N4···O1	0.82 (4)	2.27 (4)	2.788 (3)	121 (3)
N5—H1N5···S1^i^	0.80 (4)	2.84 (4)	3.522 (2)	144 (3)
N5—H2N5···O1^ii^	0.82 (3)	2.11 (4)	2.925 (3)	175 (4)

Symmetry codes: (i) *x*, −*y*+1, *z*+1/2; (ii) −*x*+1/2, *y*−1/2, −*z*+1/2.
